# Improving On Fast-track Protocol for Post Cardiac Surgery Patients

**DOI:** 10.1186/1749-8090-10-S1-A330

**Published:** 2015-12-16

**Authors:** Yi C Tham, Zhihui Tan, Anna LW Tam, Shah S Sharad, Kenny YK Sin, Shaw Y Chia, Kim K Ong

**Affiliations:** 1 Department of Cardiothoracic Surgery, National Heart Centre Singapore, Singapore, 169609, Singapore; 2 Department of Anaesthesia, Singapore General Hospital, Singapore, 169609, Singapore; 3 Department of Statistics, Singapore Cardiac Databank (National Heart Centre), Singapore, 169609, Singapore

## Background/Introduction

Many risk factors have been shown to be independently predictive of the success of fast-track for post cardiac surgery patients. While a safe fast-track protocol is important, patient selection is crucial too in determining the success of fast-tracking patients.

## Aims/Objectives

We aim to improve on our fast-track protocol by identifying risk factors affecting extubation time in our institution.

## Method

For a total of six months duration, we non-selectively included all cardiac surgery patients admitted through our new post-anaesthesia care unit (PACU). We studied how patients' profile, comorbidities and operative data affect the success rate of extubation.

## Results

107 of the total 145 patients admitted to PACU were able to be extubated. However, only 79(54.5%) patients were able to be extubated within four hours. Within the success group, we found that age (OR = 0.912; 95% CI = 0.044-1.779) and duration of ventilator weaning (OR = 0.813; 95% CI = 0.698-0.928) significantly influenced the extubation time with p-value of 0.040 and <0.001 respectively. Within the failure group, age (HR = 1.061; 95% CI = 1.018-1.105), EUROscore II (HR = 2.303; 95% CI = 1.416-3.748), cardiopulmonary bypass time (HR = 1.015; CI 95% 1.005-1.025), aortic cross-clamp time (HR = 1.023; 95% CI = 1.010-1.037) and post-operative inotropic usage (HR = 2.892; 95% CI = 1.637-5.109) significantly affect failure of extubation with p-values of 0.005, 0.001, 0.004, 0.001 and <0.001 respectively.

## Discussion/Conclusion

Through this observational study, we will be able to improve on our pre-operative patient selection based on their age and EUROscore II; and intra-operative decision based on the total cardiopulmonary bypass time and aortic cross-clamp time in order to fast-track cardiac patients by admitting them to PACU. Through this, fast-track protocol can be practiced safely to its many advantages.

## Consent

Written informed consent was obtained from the patient for publication of this abstract and any accompanying images. A copy of the written consent is available for review by the Editor of this journal.

